# Preliminary Outcomes of Temporary Collagen Punctal Plugs for Patients with Dry Eye and Glaucoma

**Published:** 2020-01-01

**Authors:** Ming Chen, So Yung Choi

**Affiliations:** 1 John A. Burns School of Medicine - University of Hawaii, Honolulu, United States

**Keywords:** Dry Eye disease, Primary Open Angle Glaucoma, Glaucoma, Collagen Punctal Plug, Punctal Closure, Intraocular Pressure

## Abstract

The aim of this study was using a temporary collagen punctal plug as a pre-test before decision of permanent punctal closure or long-term plug use for patients with dry eye disease (DED) and primary open-angle glaucoma (POAG). This case-control study was conducted at a single office in Honolulu, Hawaii from January 2017 to August 2018. In the case group, a temporary collagen plug was used in 33 eyes of 33 patients with DED and POAG who were receiving glaucoma medications with good control. On the other hand in the control group, 33 eyes of 33 patients with DED and POAG who were receiving glaucoma medications with good control were included, but punctal plug was not used. In the case group, one of the lower lid puncta was selected for the study and a canalicular rod shape plug was inserted as a therapeutic trial to predict the efficacy of long-term punctal plug placement or punctal closure. The mean changes of intraocular pressure (IOP) and improvement in symptoms/signs of DED were compared between the two study groups. Results revealed a statistically significant IOP reduction in the case group compared to the control group. Furthermore, DED improved significantly more in the case group compared to the control group (P< 0.001). We concluded that temporary punctal plug in patients with DED and POAG can significantly improve DED and lower IOP. Therefore, we could consider permanent punctal closure or long-term plug for patients with DED and POAG who responded well to temporary punctal plug without epiphora or other complications.

## INTRODUCTION

The incidence of dry eye disease (DED) has been reported as 7.4% to 33.7%, predominantly found in females and the elderly [1]. Punctal plug occlusion has been reported as an effective treatment for dry eyes by some studies [2]. Song et al. in a systematic review reported one of the misconceptions about punctal plugs reporting that they may be effective primarily in DED with aqueous deficiency. Several studies demonstrated that plugs are effective in various ocular DEDs [3]. Treatment of patients with DED and primary open-angle glaucoma (POAG) can be challenging as topical intraocular pressure (IOP) lowering medications used to treat glaucoma can also worsen signs and symptoms of DED. Matthews et al. reported that 45-60% of patients with glaucoma using topical hypotensive medications have some degrees of ocular surface disease [4].

 Punctal occlusion can improve DED and also glaucoma by increasing retention of IOP-lowering medications. Besides, punctal occlusion can decrease absorption of glaucoma medications into nasal mucosae, such as beta-blockers to prevent affecting the heart rate. One study demonstrated that punctal occlusion reduces 60% of systemic absorption of topical timolol [5]. Also, Huang et al. found a statistically significant reduction in IOP after punctal plug occlusion with a difference of 1.82 mmHg or 10.7% (baseline of 16.96 mmHg) [6]. Nonetheless, some clinical studies found questionable efficacy regarding glaucoma therapy using punctal plug [7, 8]. Although punctal plug placement is safe, epiphora, conjunctival irritation and punctal plug extrusion have been reported as most common complications. Canaliculitis and dacryocystitis, which are caused by Actinomyces* israelii*, Staphylococci, Streptococci and Diphtheroids are also infrequent [9]. Both intracanalicular plugs and external plug can be inadvertently pushed down into the canaliculus during insertion. Besides, an external plug can be lost after insertion. Therefore, a temporary fast absorbable collagen plug can be used as a pre-test before considering long-term punctal plugs or permanent closure to avoid these complications. The temporary absorbable intracanalicular collagen plug will be absorbed in two days to two weeks [10, 11].

This study aimed to investigate whether absorbable intracanalicular collagen temporary punctal plug inserted in patients with DED and POAG can improve patients’ ocular surface disease and improve the efficacy of topical IOP lowering medications without complications. 

## METHODS

This was a case-control study conducted from January 2017 to August 2018. The study protocol was approved by the institutional review committee of the University of Hawaii. All study participants gave a written informed consent. In the case group, the temporary plug was used in 33 eyes of 33 patients who had well-controlled chronic POAG and DED, while receiving IOP lowering medications such as Timolol or latanoprost. One of the lower lid puncta was selected for the study. However, in the control group, 33 eyes of 33 patients with chronic POAG and DED and receiving their IOP lowering medications such as Timolol or latanoprost were recruited, but plug was not used. Eyes with POAG were confirmed clinically by IOP above 20 mmHg, open-angle in the gonioscopic examination, visual field defect on Humphrey visual field test (Carl Zeiss Meditec, Jena, Germany) and objective findings in Optical Coherence Tomography (OCT) (Stratus-OCT Zeiss, Jena, Germany) imaging. 

The inclusion criteria were adults (50-90-years-old) with DED and well-controlled chronic POAG with IOP in normal range while using their topical IOP lowering medications. The exclusion criteria were severe and poorly controlled POAG, severe ocular surface disease, chronic dacryocystitis, nasolacrimal duct obstruction, history of lacrimal, glaucoma or refractive surgery, history of contact lens wear and those who could not adhere to follow-up at two weeks.

**Figure 1:**
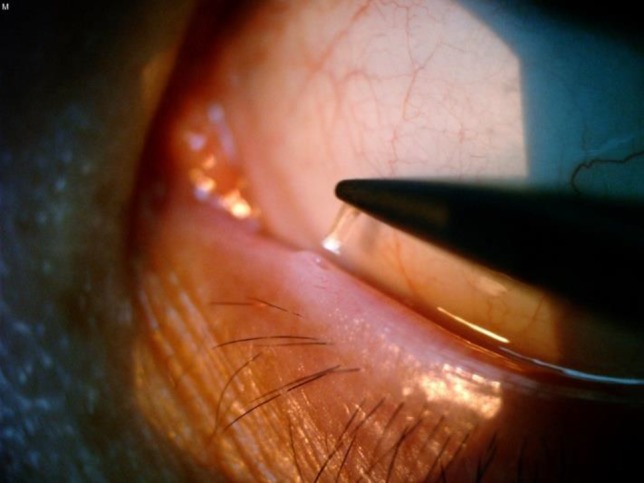
Insertion of Vertical Canalicular Soft Collagen Plug in Left Lower Punctum in Case Group

In the cases group, a canalicular rod shape plug was inserted for a temporary therapeutic trial (Figure 1) to predict long-term punctal plug placement or punctal closure. Punctal plug was selected according to the size of vertical canalicular soft collagen plug (from 0.3 to 0.5 mm diameter, Oasis, Lacrimedics, Glendora, Ca, The USA) that was easily but tightly inserted into the punctum without dilation [11]. 

**Figure 2:**
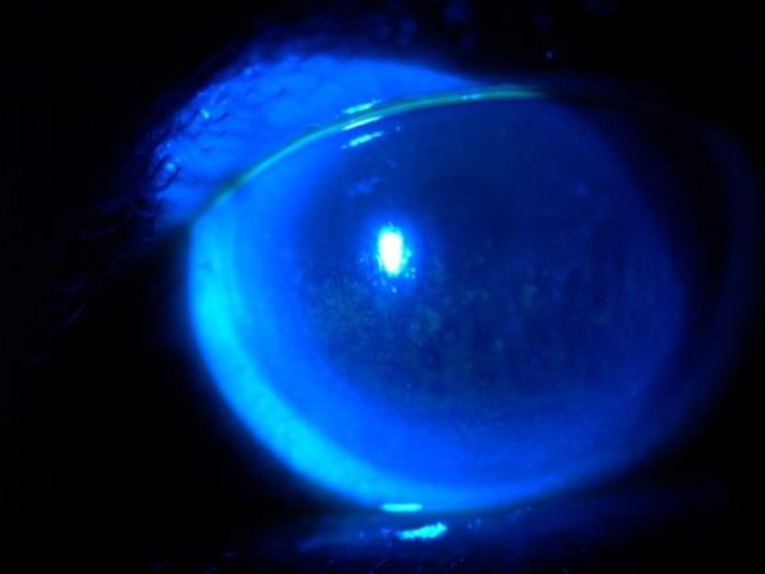
Slit-photo Under Diffuse Illumination With Blue Cobalt Light Before Insertion of the Punctual Plug in the Case Group, Showing Diffuse Punctate Epithelial Staining of the Cornea.

**Figure 3:**
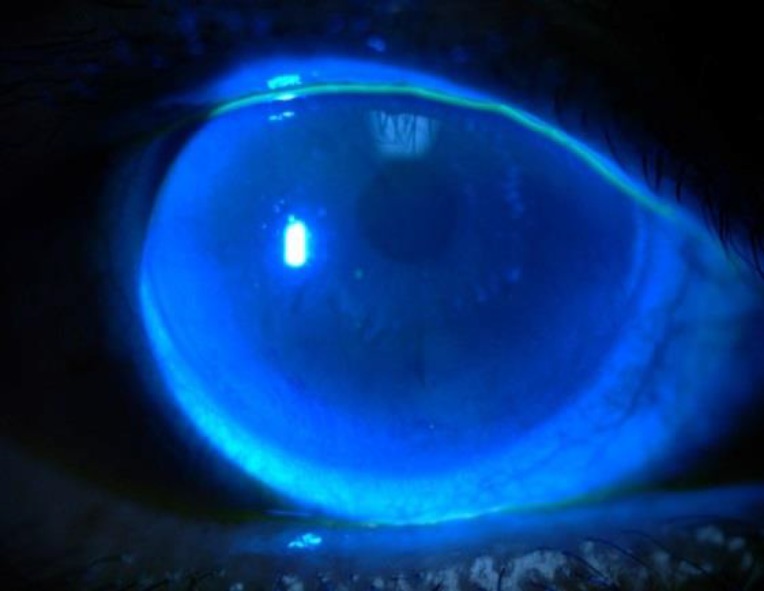
Slit-photo Under Diffuse Illumination With Blue Cobalt Light Two Weeks Following Insertion of the Punctual Plug in the Case Group, Showing Less Punctate Epithelial Staining of the Cornea.

IOP was checked by a single technician (M.C.) using a non-contact tonometer called Ocular Response Analyzer (ORA) (Reichert Ophthalmic Instruments Inc., Depew, NY) and the corneal compensated IOP (IOP-CC) considered for analysis. IOP measurement was performed at the same office visit time before and 2 weeks after a temporary punctal plug insertion in the case group and at first visit and after two weeks follow-up in the control group. Symptom improvement was checked according to the OSDI (Ocular Surface Disease Index) criteria including light sensitivity, gritty sensation, pain and blurring of vision. Moreover, TFOS DEWSI (The international Tear Film and Ocular Surface Society Dry Eye Workshop I) dry eye diagnostic criteria was used [12]. Improvement of cornea stain and tear break up time (TBUT) were assessed as the outcome measures (Figures 2-3). 

Descriptive statistics using frequency and percentage for categorical variables, and mean and standard deviation (SD) for continuous variables were calculated. Fisher’s exact test and two-sample t-test were used. All statistical tests were two-sided and p-value < 0.05 was considered as statistically significant. R software, version 3.6.0 (R Core Team, 2019) was used to analyze data.

**Table 1:** Demographic Characteristics and Final Evaluations of Case and Control Groups

Variable	Case Group Eyes Treated with Punctum Plug (n=33) n (%) or Mean ± SD	Control Group Eyes Treated without Punctum Plug (n=33) n (%) or Mean ± SD	p-value
Sex			0.999
** Male**	15 (45.45%)	14 (42.42%)	
** Female**	18 (54.55%)	19 (57.58%)	
Age	76.97 ± 7.35	75.33 ± 9.22	0.428
Location			
** E2 (OS); Left**	20 (60.61%)	Not Applied	
** E4 (OD); Right**	13 (39.39%)		
Dry Eye Disease level			
** DED 1**	1 (3.03%)	22 (66.67%)	**<0.001 **
** DED 2**	23 (69.70%)	5 (15.15%)	**<0.001 **
** DED 3**	8 (24.24%)	5 (15.15%)	0.325
** DED 4**	1 (3.03%)	1 (3.03%)	0.754
Punctum Size (mm)			
** 0.3**	2 (6.06%)	Not Applied	
** 0.4**	7 (21.21%)		
** 0.5**	24 (72.73%)		
Eyes with IOP Change			
** Decreased**	22 (66.67%) -4.18 ± 2.74	18 (54.55%) -2.94 ± 2.07	0.531
** Same**	3 (9.09%)	6 (18.18%)	0.052
** Increased**	8 (24.24%) 2.00 ± 1.41	9 (27.27%) 4.11 ± 3.55	0.522
Improvement of symptom and signs	**Less corneal staining**	**No change**	
** Same**	5 (15.15%)	31 (93.94%)	**<0.001**
** Improved**	28 (84.85%)	2 (6.06%)	**<0.001**
Mean OSDI score	20.24	30.21	0.189

Abbreviations: SD: standard deviation; n: number; %: percentage; E2: left lower lid punctum; E4: right lower lid punctum; OS: left eye; OD: right eye; DED: dry eye disease; mm: millimeter; IOP: intraocular pressure; S&S: symptom and sign; OSDI: Ocular Surface Disease Index; Case group: subjects with punctal plug; Control group: subjects without punctal plug. P<0.05 in bold.

**Table 2:** Baseline and Final Intraocular Pressure (IOP) of the Case and Control Groups

	Case Group Mean± SD	Control Group Mean± SD
IOP at First Visit; mmHg	17.09±4.58	13.94±3.42
IOP at Second Visit (2 weeks later); mmHg	14.39±3.18	13.45±3.46
IOP difference; mmHg	-2.7±3.53	-0.48±3.85

The final IOP was significantly lower in the case group (paired t-test, p-value=0.0001), yet not in the control group (paired t-test; p-value=0.4746). Abbreviations: SD: standard deviation; mmHg: millimeters of mercury; Case group: subjects with punctal plug; Control group: subjects without punctal plug.

## RESULT

Thirty-three eyes of 33 patients in the case and 33 eyes of 33 patients in the control group with the mean ± SD age of 76.97 ± 7.35 and 75.33 ± 9.22 years, respectively were evaluated (Table 1). The two groups were age-sex matched with P-values of 0.428 and 0.999, respectively. In the case group, 20 (60.61%) eyes had punctal plug of the left lower lid and 13 (39.39%) in the right lower lid (Table 1). According to the severity of DED in each study group, patients were divided into four subgroups including DED 1, DED 2, DED 3 and DED 4. In case and control groups, 1 (3.03%) and 22 (66.67%) eyes considered as DED 1, 23 (69.70%) and 5 (15.15%) eyes as DED 2, 8 (24.24%) and 5 (15.15%) eyes as DED 3, and 1 (3.03%) and 1 (3.03%) eye as DED 4, respectively. The number of patients with DED 1 and DED 2 in the case group was significantly different from the control group (P<0.001). Punctal diameter in the case group were as follows; in 24 (72.73%) eyes was 0.5 mm, in 7 (21.21%) eyes 0.4 mm and in 2 (6.06%) eyes 0.3 mm (Table 1). Evaluating signs and symptoms of DED two weeks after the study showed that in the case group 28 eyes (84.85%) and in the control group only two eyes (6.06%) had improvement, with a statistically significant difference. Also, the case group had a lower OSDI score compared to the control group, which indicates improvement in ocular surface disease index in the case group (20.24 versus 30.21) (Table 1). At the end of study, we evaluated changes in IOP between the two groups as well as between baseline and final IOP in each group. Although, the number of eyes did not differ significantly between the groups with decreasing IOP, increasing IOP and constant IOP (Table 1). However as shown in Table 2, the final IOP in the case group was significantly lower than the baseline (P=0.001), but not in the control group (P=0.474).

## DISCUSSION

In the current study, we found that temporary collagen punctum plug can reduce IOP and improve symptoms and signs of DED without the risk of permanent punctum closure. We intended to develop this test to avoid irreversible complications from permanent punctal closure.

 It is well known that punctal plug can improve DED and reduce IOP in patients with glaucoma [3, 6]. Sherwin et al. also indicated the efficacy of punctal plug to control IOP and improve DED. However, they inserted a pre-loaded silicone punctal plug [13], whereas in the current study we used a vertical canalicular soft collagen plug. The silicon plug is more expensive, irritable and with the possibility of loss after insertion. Also, permanent punctal plug may cause irreversible epiphora [9]. A short-term economical collagen plug may be an option before placing permanent or semi-permanent silicone plugs. Collagen absorbable plugs are made of bovine collagen, which is generally well-tolerated. However, approximately 3% of population is allergic to bovine collagen [14, 15]. Ahn et al. reported a case of canaliculitis three years after the insertion of plug [15]. Mullins reported two cases with allergic reaction to bovine collagen [16]. Fortunately in our study, no allergic reaction was reported. 

Patients in our trial were receiving treatment for both DED and glaucoma during the study, therefore, the validity of our results may be affected by some confounding factors such as compliance, age, other comorbidities and finance [17]. However, the result of this study matched previous studies using punctal plug [2, 6]. Totally, 45-60% of patients with glaucoma using topical IOP lowering medications have an ocular surface disease, therefore clinicians must consider punctal plug as one of the adjunct treatment for these patients. Some studies have reported that plugs are effective in a variety of ocular diseases, not just aqueous deficiency DED [3]. 

This was the first study regarding temporary collagen punctal plug on lowering IOP and improving DED. Before placing an expensive silicon plug or permanent thermal punctal closure, an irreversible tearing from punctal closure or wasting an expensive non-effective silicon plug must be kept in mind. Here, a non-expensive temporary collagen plug was used to predict the efficacy of long-term ones and assess possible complications.The limitations of this study were its single-center design by a single physician, short time follow-up and not using the counterpart eye of each subject as the control group. The strength of this study was unified observation by a single physician and single automatic tonometry for IOP check. Further multicenter prospective double-blind studies may be needed to confirm the confidentiality of findings.

## CONCLUSION

A temporary collagen punctal plug could improve DED and reduce IOP. In those eyes that responded to temporary plug without complications, a long-term punctal plug or permanent punctal closure could be considered. 
